# Amphiregulin/epidermal growth factor receptor/hypoxia-inducible factor-1α pathway regulates T helper 9 and T cytotoxic 9 cell response in adult patients with infectious mononucleosis

**DOI:** 10.17305/bjbms.2022.8013

**Published:** 2023-01-06

**Authors:** Yu Li, Lan Li, Weihua Zhang, Ying Gao

**Affiliations:** 1 Department of Infectious Diseases, Shaanxi Provincial People’s Hospital, The Affiliated Hospital of Xi’an Medical University, Xi’an, Shaanxi Province, China; 2 Department of Hematology, Shaanxi Provincial People’s Hospital, The Affiliated Hospital of Xi’an Medical University, Xi’an, Shaanxi Province, China

**Keywords:** Infectious mononucleosis (IM), T helper 9 (Th9) cells, T cytotoxic 9 (Tc9) cells, amphiregulin (AREG), growth factor receptor, hypoxia-inducible factor-1α (HIF-1α)

## Abstract

Amphiregulin (AREG)/epidermal growth factor receptor (EGFR) signaling induces hypoxia-inducible factor-1α (HIF-1α), leading to promotion of T helper 9 (Th9) differentiation and anti-tumor functions. However, the role of the AREG/EGFR/HIF-1α pathway in regulating interleukin-9 (IL-9) production by T cells in adult patients with infectious mononucleosis (IM) has not been fully elucidated. Fifty IM patients and 20 controls were enrolled. The percentages of Th9 and T cytotoxic 9 (Tc9) cells, the mRNA relative expressions of the transcription factors of IL-9-secreting T cells, purine-rich nucleic acid binding protein 1 (PU.1) and forkhead box protein O1 (FOXO1), and the levels of IL-9, AREG, EGFR, and HIF-1α were measured. Peripheral blood mononuclear cells from IM patients were stimulated with EGFR inhibitor or exogenous AREG in the presence or absence of anti-HIF-1α. Regulation of the AREG/EGFR/HIF-1α pathway to IL-9 production by T cells was assessed. The percentages of Th9 and Tc9 cells, plasma IL-9 levels, and *PU.1* and *FOXO1* mRNA expressions were elevated in IM patients. Plasma levels of AREG and HIF-1α were also increased in IM patients. AREG levels correlated positively with the percentages of Th9 and Tc9 cells in IM patients. Inhibition of EGFR suppressed IL-9-producing T cell differentiation and HIF-1α production. Exogenous AREG stimulation not only induced EGFR and HIF-1α expression but also promoted IL-9-secreting T cell differentiation. Neutralization of HIF-1α abrogated AREG/EGFR-induced Th9 and Tc9 differentiation in IM patients. The current data suggested that the AREG/EGFR/HIF-1α pathway contributed to the elevation of Th9 and Tc9 differentiation in IM patients.

## Introduction

Infectious mononucleosis (IM) is a disease mainly caused by Epstein–Barr virus (EBV) infection [1]. Infection with EBV during childhood is usually asymptomatic or mild in the majority of individuals [2]. However, viral genome remains latent in adolescents and young adults, leading to the establishment of lifelong persistent infection in a small portion of memory B cells [3]. Adolescent and young adult IM patients typically manifest with fatigue, fever, pharyngitis, and lymphadenopathy [4]. Treatment of IM patients includes anti-EBV and supportive therapy. However, no antiviral agent has been confirmed for treatment of EBV infection and clinical efficacy is limited [5]. Importantly, the pathogenesis of IM is not fully understood, which limits the development of antiviral strategies for EBV-induced IM patients.

IM patients have higher peripheral CD3^+^, CD4^+^, and CD8^+^ T lymphocyte cell count [6]. However, EBV-specific CD4^+^ and CD8^+^ T cell responses are low and remain similarly consistent even six months after diagnosis of IM [7]. CD4^+^ T cells can be distinguished into different T helper (Th) subpopulations by various transcription factors initiation and the cytokines induction [8, 9]. CD4^+^ T cells that produce interleukin-9 (IL-9) are defined as T helper 9 (Th9) cells, and they can be induced by transcription factor purine-rich nucleic acid binding protein 1 (PU.1) and forkhead box protein O1 (FOXO1) [10, 11]. CD8^+^ T cells that secrete IL-9 are defined as T cytotoxic 9 (Tc9) cells [12]. Both Th9 and Tc9 cells contribute to parasitic infection [13, 14] and tumor immunity [15, 16]. However, the regulation of Th9 and Tc9 response in both physiological and pathological conditions is not completely elucidated.

Epidermal growth factor receptor (EGFR) is an Erb-B family member, which is expressed on both epithelial and immune cells [17]. EGFR is activated through binding of its ligands, including epidermal growth factor (EGF), transforming growth factor α (TGF-α), and amphiregulin (AREG), leading to the phosphorylation of tyrosine kinase domain [18]. EGFR signaling also induces the activation of hypoxia-inducible factor-1α (HIF-1α) in cancers [19], which plays an important role in Th cell differentiation and function [20]. A more recent study by Roy et al. [21] demonstrated that the AREG/EGFR pathway mediates HIF-1α, which transactivates IL-9 promoters and promotes anti-tumor activity of Th9 cells. Thus, the AREG/EGFR/HIF-1α signaling pathway contributes to Th9 cell differentiation and anti-tumor function. However, few reports have focused on the function and modulation of IL-9-producing T cells in IM patients. Although IL-9 was sporadically detectable in EBV positive non-nasal peripheral T cell lymphoma [22], expression of IL-9 mRNA was strongly elevated in nasal natural killer T-cell lymphoma cell lines and patients, which is closely associated with EBV infection [23]. In this study, we investigated Th9 and Tc9 cells in IM patients. The regulatory function of the AREG/EGFR/HIF-1α pathway to IL-9-secreting T cells in IM patients was then assessed in vitro.

## Materials and methods

### Studied subjects

Fifty adult IM patients were enrolled in the present study. Inclusion criteria were: (1) Age >18 years old when admitted. (2) Meeting the IM diagnostic criteria: 2a) Clinical indicators (meeting three or more): fever, pharyngeal tonsillitis, cervical lymphadenopathy, splenomegaly, hepatomegaly, eyelid edema; 2b) Laboratory indicators (meeting one or more): (a) Positive for EBV DNA in the peripheral blood; (b) Positive for anti-EBV-viral capsid antigen-IgM and IgG, while negative for anti-EBV nuclear antigen-IgG; (c) More than four times elevation of double serum anti-EBV-viral capsid antigen-IgG titer; (d) Atypical lymphocyte ratio in peripheral blood ≥ 0.10 and/or lymphocytosis 5.0×10^9^/L. Exclusion criteria were: (1) Other chronic viral infection; (2) Cancer; (3) Autoimmune diseases; (4) Severe systemic or organic failure, such as liver or renal failure. Meanwhile, 20 healthy individuals with matched sex ratio and average age were also included in the study as controls. Participants’ sex was defined based on self-report. The sample size numbers were calculated by Clinical Research Sample Size Calculator.

### Plasma and peripheral blood mononuclear cells isolation

Twenty milliliters of EDTA anticoagulant peripheral blood was obtained from all study subjects. Plasma was obtained by centrifugation at 1000×*g* for 10 min. Peripheral blood mononuclear cells (PBMCs) were isolated by using Ficoll-paque Premium (GE Healthcare Bio-Science AB, Bjokgatan, Uppsala, Sweden) as previously described [24]. Briefly, the whole blood was diluted by 15 mL of phosphate buffered saline (PBS) buffer. Fifteen milliliters of Ficoll-paque Premium were carefully layered over of diluted blood. The mixture was centrifuged at 400×*g* for 30 min at 20 ^∘^C in a swing bucket rotor with no brake. The cloudy mononuclear cell layer was carefully transferred to a fresh tube and washed twice with PBS. PBMCs were cultured with RPMI 1640 medium supplemented with 10% fetal bovine serum, penicillin (100 U/L), and streptomycin (0.1 mg/mL) under 5% CO_2_ condition.

### Cell culture

5×10^5^ of PBMCs from 16 randomly selected IM patients were stimulated with gefitinib (R&D Systems, Minneapolis, MN, USA; final concentration: 1 µg/mL), which inhibits tyrosine kinase activity of EGFR through binding to the adenosine triphosphate-binding domain [25], for 72 h. 5×10^5^ of PBMCs from 13 randomly selected IM patients were stimulated with AREG (the ligand for EGFR) (R&D Systems, Minneapolis, MN, USA; final concentration: 100 ng/mL) [21] in the presence or absence of anti-HIF-1α (R&D Systems, Minneapolis, MN, USA; Clone #241809; final concentration: 5 µg/mL) for 72 h. Gefitinib is an EGFR inhibitor that interrupts signaling by EGFR in target cells. AREG is a member of the EGF family. AREG interacts with the EGF/TGF-α receptor to promote the growth of normal epithelial cells and to inhibit the growth of certain aggressive carcinoma cell lines.

### Flow cytometry

PBMCs were stimulated with phorbol myristate acetate (50 ng/mL) and ionomycin (1 µg/mL) in the presence of brefeldin A (10 µg/mL) for 6 h. Cells were stained with anti-CD3-fluorescein isothiocyanate (BD Pharmingen, San Jose, CA, USA; Clone UCHT1), anti-CD4–peridinin–chlorophyll–protein complex (BD Pharmingen, San Jose, CA, USA; Clone SK3), anti-CD8–phycoerythrin (BD Pharmingen, San Jose, CA, USA; Clone HIT8α) for 30 min in the dark at 4 ^∘^C. Cells were then fixed and permeabilized using BD Cytofix/Cytoperm Fixation/Permeabilization (BD Biosciences, San Jose, CA, USA) and stained with anti-IL-9–allophycocyanin (R&D Systems, Minneapolis, MN, USA; Clone #623153). Flow cytometric analysis was performed using a BD LSR II System (BD Biosciences, San Jose, CA, USA).

### Enzyme-linked immunosorbent assay

IL-9, EGFR, AREG, and HIF-1α levels were measured by commercial enzyme-linked immunosorbent assay (ELISA) kits purchased from R&D Systems (Minneapolis, MN, USA), including Human IL-9 DuoSet ELISA (Catalog Number: DY209-05), Human EGFR DuoSet ELISA (Catalog Number: DY231), Human Amphiregulin DuoSet ELISA (Catalog Number: DY262), and Human/Mouse Total HIF-1 alpha/HIF1A DuoSet IC ELISA (Catalog Number: DYC1935-2). Briefly, 100 µL of samples or standards were added to the wells of plates and incubated for 2 h at room temperature. The plates were washed five times. One hundred microliters of the detection antibodies were added to each well and incubated for 2 h at room temperature. The plates were washed five times. One hundred microliters of streptavidin-horseradish peroxidases were added to each well and incubated at room temperature for 20 min. The plates were washed five times. One hundred microliters of substrate solutions were added to each well and incubated at room temperature for 20 min. Fifty microliters of stop solutions were added to each well. The optical density of each well was determined using a microplate reader set to 450 nm.

### Real-time quantitative reverse transcription polymerase chain reaction

Total RNA was extracted using TRIzol reagent (Invitrogen, Carlsbad, CA, USA). cDNA was synthesized using PrimeScript RT Master Mix (TaKaRa, Beijing, China).

Reverse transcriptional reaction system contained 5×PrimeScript RT Master Mix (Perfect Real Time) 2 µL, total RNA 1 µg, and RNase free dH_2_O up to 10 µL. Reverse transcriptional reaction protocol was 37 ^∘^C for 15 min, 85 ^∘^C for 5 s. Real-time PCR was performed using TB Green Premix Ex Taq (TaKaRa, Beijing, China). PCR reaction system contained: 2×TB Green Premix Ex Taq II (Tli RNaseH Plus) 25 µL, PCR forward primer (10 µmol/L) 2 µL, PCR reverse primer (10 µmol/L) 2 µL, cDNA solution 4 µL, and ddH_2_O 16 µL. PCR reaction protocol was 95 ^∘^C 30 s for 1 cycle, 95 ^∘^C 5 s, 60 ^∘^C 30 s for 40 cycles. The target gene levels (including *PU.1*, *FOXO1*, *HIF-1*α, and *EGFR*) were relatively quantified using comparative Ct method formula 2^−ΔΔCt^ on ABI7500 Sequence Detector System (Applied Biosystems, Foster, CA, USA). The primer sequences were cited from previously published literature [12, 26, 27]. *PU.1* forward primer: 5’-GGA AGC CCG GCT GGA TGT TAC-3’, *PU.1* reverse primer: 5’-CAC CAG GTC TTC TGA TGG CTG A-3’; *FOXO1* forward primer: 5’-ACA GAC CAA CCT GGC ATT AC-3’, *FOXO1* reverse primer: 5’-TAC GTC CTG ATG GGA CTT ACA-3’; *HIF-1*α forward primer: 5’-CCC ATT CCT CAC CCA TCA AAT A-3’, *HIF-1*α reverse primer: 5’-CTT CTG GCT CAT ATC CCA TCA A-3’; *EGFR* forward primer: 5’-GAC AGG CCA CCT CGT CG-3’, *EGFR* reverse primer: 5’-TCG TGC CTT GGC AAA CTT TC-3’.

### Ethical statement

The study protocol was in accordance with the Declaration of Helsinki and was approved by the Ethics Committee of Shaanxi Provincial People’s Hospital (No. 2017019). Written consent was obtained from each enrolled individual.

### Statistical analysis

All data were analyzed using SPSS version 23.0 for Windows (Chicago, IL, USA). Shapiro–Wilk test was used for normal distribution assay. Variables following normal distribution were presented as mean ± standard deviation, and statistical significance was determined by Student’s *t* test, paired *t* test, one-way analysis of variance (ANOVA), or Tukey test. Variables following skewed distribution were presented as median and interquartile range (IQR), and statistical significance was determined by Mann–Whitney test, Wilcoxon paired test, Kruskal–Wallis test, or Dunn’s multiple comparison test. Pearson or Spearman correlation analysis was performed for correlation analysis. *P*  values of less than 0.05 were considered to indicate significant differences.

## Results

### Characteristics of participants

The clinical characteristics of studied subjects are shown in Table 1. Fifty IM patients and 20 controls with matched sex ratio and mean age were enrolled in the study. Lymphocytes count was significantly increased in IM patients compared to controls (*P* < 0.0001). Neither atypical lymphocytes nor EBV DNA were detected in controls. Twenty-nine (58%) IM patients were positive for EBV DNA in the peripheral blood.

**Table 1:** The clinical characteristics of studied subjects

	**Controls**	**IM patients**
Cases (*n*)	20	50
Sex (male/female)	11/9	28/22
Age (years)	30.10 ± 8.76	27.92 ± 7.33
Lymphocytes (×10^9^/L)	1.66 ± 0.56	4.67 ± 1.16
Atypical lymphocyte ratio	Not available	0.12 ± 0.02
EBV DNA positive (*n*, %)	Not available	29 (58)

EBV: Epstein–Barr virus; IM: Infectious mononucleosis.

### Th9 and Tc9 percentages were elevated in IM patients

**Figure 1.**
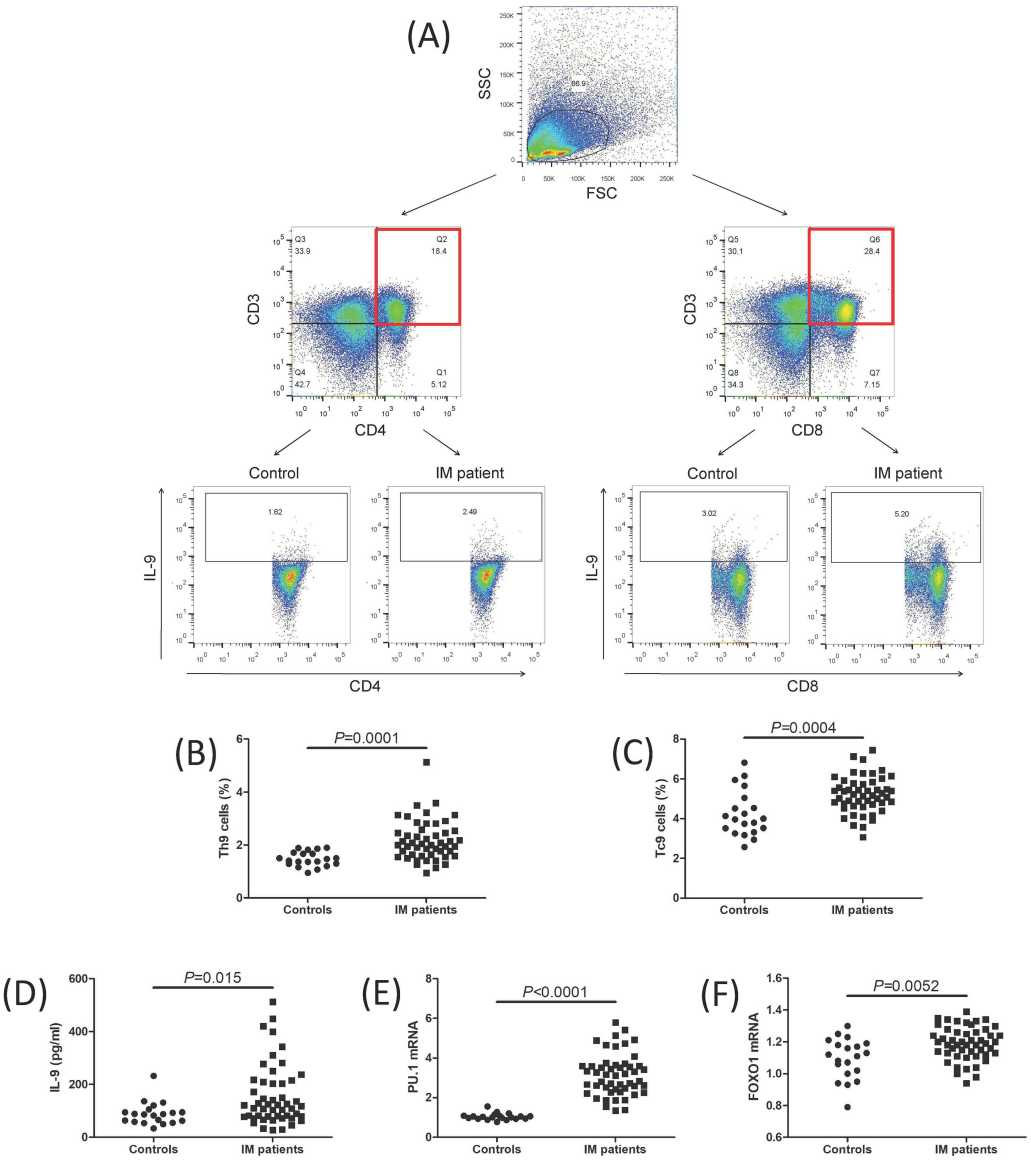
**Th9 and Tc9 cell percentage, IL-9 levels, and transcription factor mRNA relative levels in IM patients.** (A) The flow cytomic analysis for Th9 and Tc9 cells in control and IM patient. Peripheral blood mononuclear cells were stained with anti-CD3, anti-CD4, anti-CD8, and anti-IL-9. CD3^+^CD4^+^IL-9^+^ cells were defined as Th9 cells, while CD3^+^CD8^+^IL-9^+^ cells were defined as Tc9 cells. (B) Th9 percentage was compared between controls and IM patients. (C) Tc9 percentage was compared between controls and IM patients. (D) Plasma IL-9 levels were measured by ELISA and were compared between controls and IM patients. Transcription factors for Th9 and Tc9, including (E) *PU.1* and (F) *FOXO1* mRNA relative levels, were relatively quantified by qRT-PCR, and were compared between controls and IM patients. Individual level for each subject was shown. Student’s *t* test or Mann–Whitney test was used for comparison. Th9: T helper 9 cells; Tc9: T cytotoxic 9 cells; IL-9: Interleukin 9; PU.1: Purine-rich nucleic acid binding protein 1; FOXO1: Forkhead box O1; IM: Infectious mononucleosis; ELISA: Enzyme-linked immunosorbent assay; qRT-PCR: Real-time quantitative reverse transcription polymerase chain reaction.

The representative flow cytomic analysis for Th9 and Tc9 is shown in Figure 1A. CD3^+^CD4^+^-secreting IL-9 cells were defined as Th9 cells, while CD3^+^CD8^+^-secreting IL-9 cells were defined as Tc9 cells (Figure 1A). Th9 cell percentage was increased in IM patients compared to controls (2.18 ± 0.76% vs 1.47 ± 0.28%; Student’s *t* test, *P* ═ 0.0001, Figure 1B). Tc9 percentage was also elevated in IM patients compared to controls (5.22 ± 0.91% vs 4.25 ± 1.15%, Student’s *t* test, *P* ═ 0.0004, Figure 1C). IL-9 levels in the plasma were upregulated in IM patients compared to controls (median 86.52 [IQR 59.50, 102.7]) pg/mL vs median 119.7 [IQR 77.76, 210.2] pg/mL, Mann–Whitney test, *P* ═ 0.015, Figure 1D). Transcription factors for Th9 and Tc9 cells, including PU.1 and FOXO1, were increased in IM patients compared with controls (Student’s *t* tests, *P* < 0.0001 and *P* ═ 0.0052, respectively, Figure 1E and 1F). There were no significant differences of Th9 cell percentage, Tc9 cell percentage, or plasma IL-9 levels between EBV positive and EBV negative IM patients (Figure S1A– S1C). Lymphocyte count did not significantly correlate with Th9 cell percentage, Tc9 cell percentage, or plasma IL-9 levels in IM patients (Figure S1D– S1F).

### AREG and HIF-1α levels in the plasma were increased in IM patients

**Figure 2.**
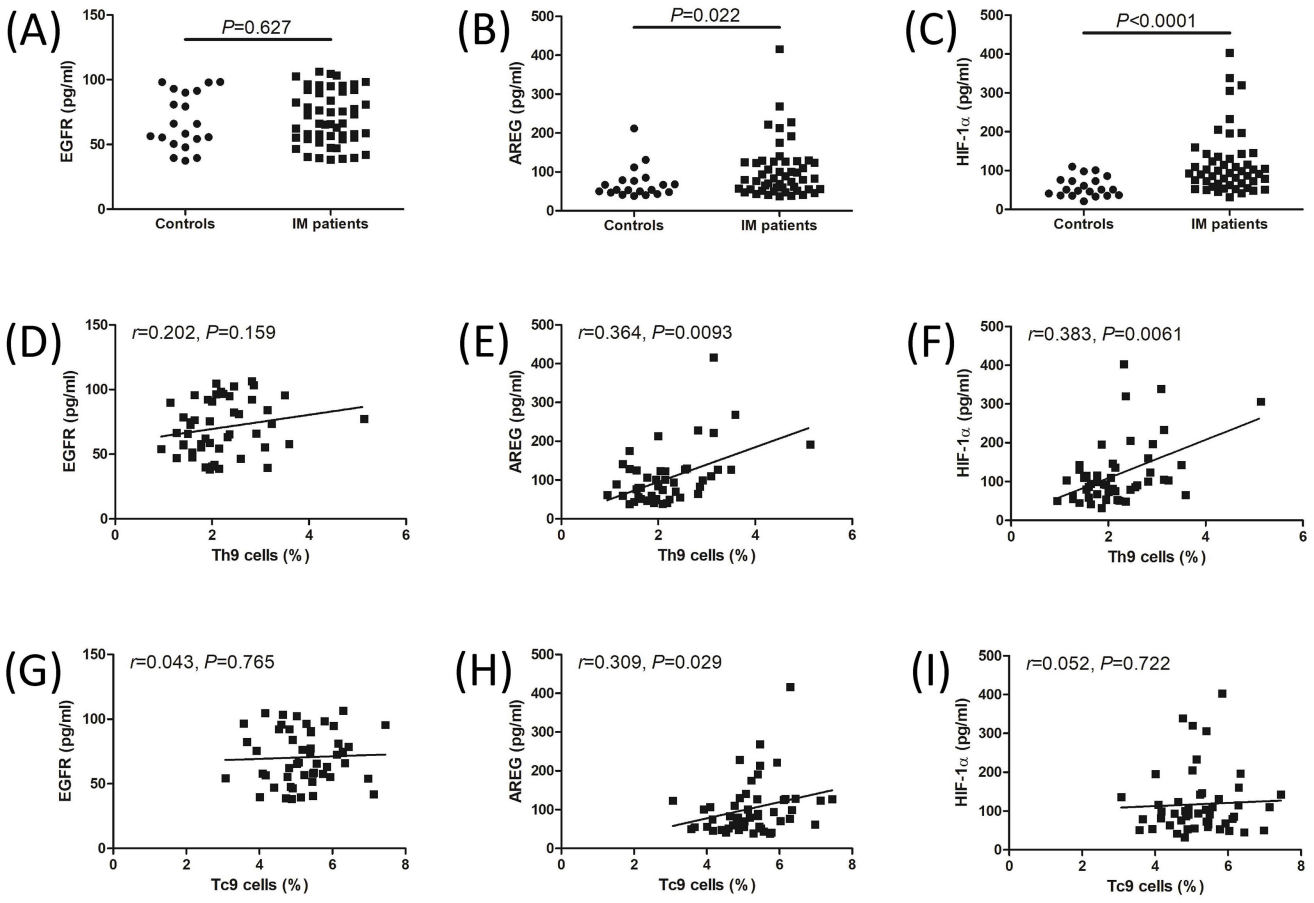
**EGFR, AREG, and HIF-1α levels in IM patients.** AREG, EGFR, and HIF-1α levels in the plasma were measured by ELISA. (A) Plasma EGFR levels were compared between controls and IM patients. (B) Plasma AREG levels were compared between controls and IM patients. (C) Plasma HIF-1α levels were compared between controls and IM patients. (D) Correlation between EGFR levels and Th9 cell percentage in IM patients. (E) Correlation between AREG levels and Th9 cell percentage in IM patients. (F) Correlation between HIF-1α levels and Th9 cell percentage in IM patients. (G) Correlation between EGFR levels and Tc9 cell percentage in IM patients. (H) Correlation between AREG levels and Tc9 cell percentage in IM patients. (I) Correlation between HIF-1α levels and Tc9 cell percentage in IM patients. Individual level for each subject was shown. Student’s *t* test or Mann–Whitney test was used for comparison. Pearson or Spearman correlation analysis was performed for correlation analysis. ELISA: Enzyme-linked immunosorbent assay; EGFR: Epidermal growth factor receptor; AREG: Amphiregulin; HIF-1α: Hypoxia-inducible factor-1α; IM: Infectious mononucleosis; Th9: T helper 9 cells; Tc9: T cytotoxic 9 cells.

There was no significant difference in EGFR levels in the plasma between controls and IM patients (67.79 ± 21.37 pg/mL vs 70.48 ± 20.59 pg/mL, Student’s *t* test, *P* ═ 0.627, Figure 2A). Plasma AREG levels were increased in IM patients compared to controls (median 81.52 [IQR 55.68, 127.9] pg/mL vs median 54.80 [IQR 47.12, 78.19] pg/mL, Mann–Whitney test, *P* ═ 0.022, Figure 2B). HIF-1α levels in the plasma were also elevated in IM patients compared to controls (median 93.85 [IQR 65.86, 137.4] pg/mL vs median 51.15 [IQR 36.52, 75.70] pg/mL, Mann–Whitney test, *P* < 0.0001, Figure 2C). There was no remarkable correlation between EGFR levels and Th9/Tc9 frequency in IM patients (*P* > 0.05, Pearson correlation analysis, Figure 2D and 2G). AREG levels positively correlated with Th9 cell percentage (*r* ═ 0.364, *P* ═ 0.0093, Spearman correlation analysis, Figure 2E) and Tc9 cell percentage (*r* ═ 0.309, *P* ═ 0.029, Spearman correlation analysis, Figure 2H) in IM patients. Plasma HIF-1α expression positively correlated with Th9 cell percentage in IM patients (*r* ═ 0.383, *P* ═ 0.0061, Spearman correlation analysis, Figure 2F). However, HIF-1α did not significantly correlate with Tc9 cell frequency in IM patients (*r* ═ 0.052, *P* ═ 0.722, Spearman correlation analysis, Figure 2I).

### Inhibition of EGFR signaling suppressed Th9 and Tc9 cells in IM patients

**Figure 3.**
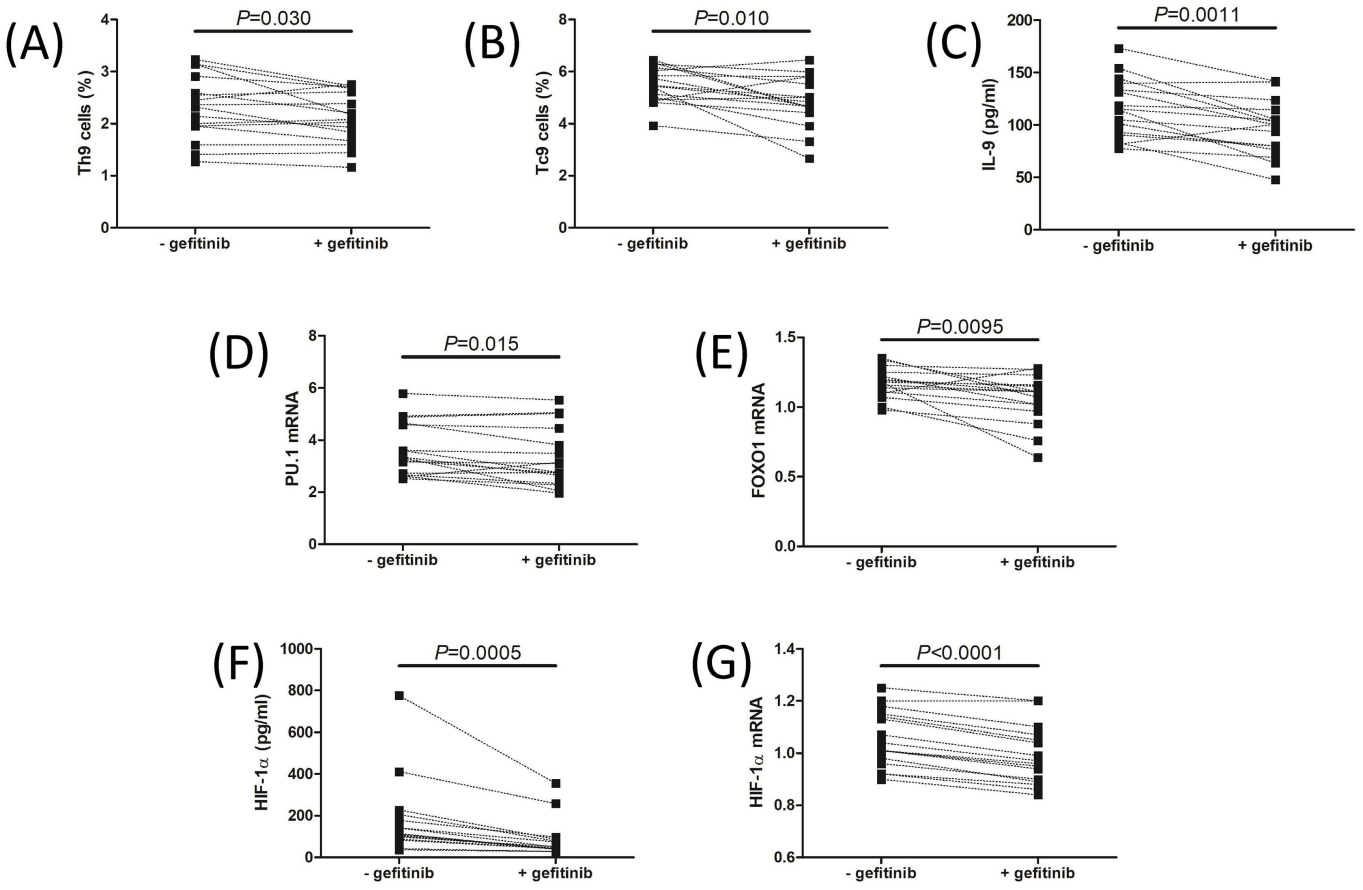
**Regulation of Th9 and Tc9 cells by inhibition of EGFR signaling.** 5×10^5^ of PBMCs from 16 IM patients were stimulated with gefitinib (1µg/mL), an inhibitor of EGFR signaling, for 72 h. (A) Th9 cell percentage and (B) Tc9 cell percentage was assessed by flow cytometry and was compared between cells with and without gefitinib stimulation. (C) IL-9 levels in the cultured supernatants were measured by ELISA and were compared between cells with and without gefitinib stimulation. Transcription factors for Th9 and Tc9, including (D) *PU.1* and (E) *FOXO1* mRNA relative levels, were relatively quantified by qRT-PCR, and were compared between cells with and without gefitinib stimulation. (F) HIF-1α levels in the cultured supernatants were measured by ELISA and were compared between cells with and without gefitinib stimulation. (G) HIF-1α mRNA relative levels in PBMCs were relatively quantified by qRT-PCR and were compared between cells with and without gefitinib stimulation. Individual level for each subject was shown. Paired *t* test or Wilcoxon paired test was used for comparison. ELISA: Enzyme-linked immunosorbent assay; PBMC: Peripheral blood mononuclear cells; qRT-PCR: Real-time quantitative reverse transcription polymerase chain reaction; EGFR: Epidermal growth factor receptor; HIF-1α: Hypoxia-inducible factor-1α; IM: Infectious mononucleosis; PU.1: Purine-rich nucleic acid binding protein 1; FOXO1: Forkhead box O1; IL-9; Interleukin 9; IM: Infectious mononucleosis; Th9: T helper 9 cells; Tc9: T cytotoxic 9 cells.

Blockade of EGFR signaling by gefitinib significantly downregulated Th9 percentage (2.12 ± 0.50% vs 2.31 ± 0.61%, paired *t* test, *P* ═ 0.030, Figure 3A) and Tc9 percentage (4.83 ± 0.98% vs 5.49 ± 0.69%, paired *t* test, *P* ═ 0.010, Figure 3B). Similarly, IL-9 production in the cultured supernatants was also reduced in response to gefitinib stimulation (96.54 ± 26.44 pg/mL vs 116.0 ± 28.16 pg/mL, paired *t* test, *P* ═ 0.0011, Figure 3C). *PU.1* mRNA and *FOXO1* mRNA relative levels were also downregulated in PBMCs with gefitinib stimulation (paired *t* tests, *P* < 0.05, Figure 3D and 3E). Importantly, gefitinib stimulation strongly inhibited HIF-1α production median 45.56 [IQR 41.76, 84.69] pg/mL vs median 112.3 [IQR 89.57, 198.6] pg/mL, Wilcoxon paired test, *P* ═ 0.0005, Figure 3F] and *HIF-1*α mRNA relative levels in PBMCs (0.99 ± 0.11 vs 1.05 ± 0.11, paired *t* test, *P* < 0.0001, Figure 3G). However, AREG production in cultured supernatants could be detected with or without gefitinib stimulation.

### AREG promoted Th9 and Tc9 cells in IM patients, which was dependent on HIF-1α production

**Figure 4.**
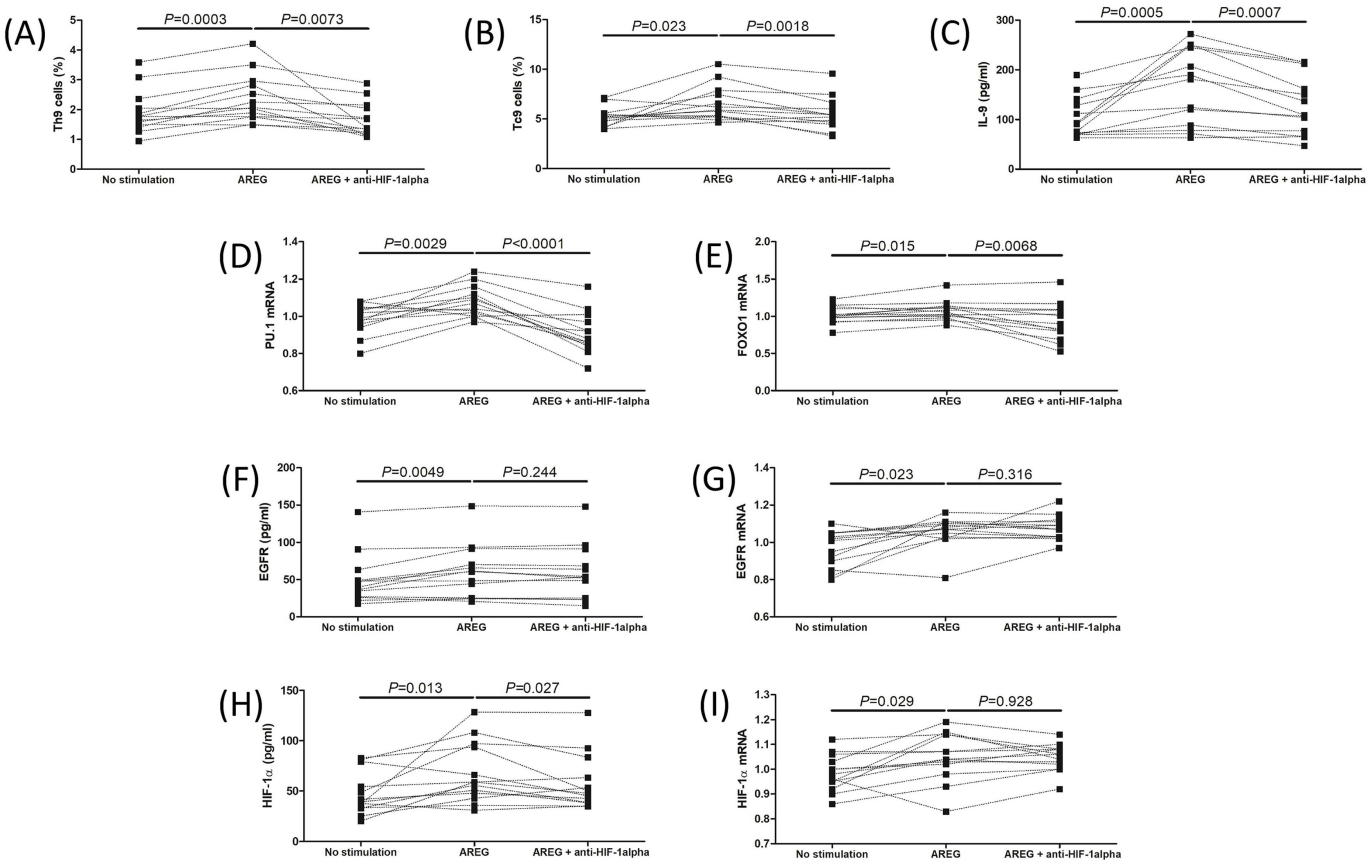
**Regulation of Th9 and Tc9 cells by exogenous AREG was dependent on HIF-1α secretion.** 5×10^5^ of PBMCs from 13 IM patients were stimulated with AREG (100 ng/mL) in the presence or absence of anti-HIF-1α (5 µg/mL) for 72 h. (A) Th9 cell percentage and (B) Tc9 cell percentage was assessed by flow cytometry and compared among cells with no stimulation, AREG stimulation, and AREG + anti-HIF-1α stimulation. (C) IL-9 levels in the cultured supernatants were measured by ELISA and compared among cells with no stimulation, AREG stimulation, and AREG + anti-HIF-1α stimulation. Transcription factors for Th9 and Tc9, including (D) *PU.1* and (E) *FOXO1* mRNA relative levels, were relatively quantified by qRT-PCR and compared among cells with no stimulation, AREG stimulation, and AREG + anti-HIF-1α stimulation. (F) EGFR levels in the cultured supernatants were measured by ELISA and compared among cells with no stimulation, AREG stimulation, and AREG + anti-HIF-1α stimulation. (G) *EGFR* mRNA relative levels in PBMCs were relatively quantified by qRT-PCR and compared among cells with no stimulation, AREG stimulation, and AREG + anti-HIF-1α stimulation. (H) HIF-1α levels in the cultured supernatants were measured by ELISA and compared among cells with no stimulation, AREG stimulation, and AREG + anti-HIF-1α stimulation. (I) HIF*-1*α mRNA relative levels in PBMCs were relatively quantified by qRT-PCR and compared among cells with no stimulation, AREG stimulation, and AREG + anti-HIF-1α stimulation. Individual level for each subject was shown. One-way analysis of variance followed with Tukey tests; Kruskal–Wallis tests followed with Dunn’s multiple comparison tests were used for comparison. ELISA: Enzyme-linked immunosorbent assay; PBMC: Peripheral blood mononuclear cells; qRT-PCR: Real-time quantitative reverse transcription polymerase chain reaction; EGFR: Epidermal growth factor receptor; AREG: Amphiregulin; HIF-1α: Hypoxia-inducible factor-1α; IM: Infectious mononucleosis; PU.1: Purine-rich nucleic acid binding protein 1; FOXO1: Forkhead box O1; IL-9: Interleukin 9; IM: Infectious mononucleosis; Th9: T helper 9 cells; Tc9: T cytotoxic 9 cells.

Exogenous AREG stimulation in vitro notably elevated Th9 percentage (2.37 ± 0.82% vs 1.91 ± 0.73%, one-way ANOVA followed with Tukey test, *P* ═ 0.0003, Figure 4A) and Tc9 percentage (6.52 ± 1.78% vs 5.22 ± 0.97%, one-way ANOVA followed with Tukey test, *P* ═ 0.023, Figure 4B). Administration of anti-HIF-1α suppressed AREG-induced Th9 cell percentage (1.67 ± 0.58%) and Tc9 cell percentage (5.52 ± 1.67%) (Tukey tests, *P* < 0.01, Figure 4A and 4B). IL-9 production in the cultured supernatants was increased in response to AREG stimulation (median 181.5 [IQR 83.35, 246.9] pg/mL vs median 90.73 [IQR 70.69, 135.0] pg/mL, Kruskal–Wallis test followed with Dunn’s multiple comparison test, *P* ═ 0.0005, Figure 4C), while anti-HIF-1α inhibited AREG-mediated IL-9 secretion (median 108.8 [IQR 71.19, 187.1] pg/mL, Dunn’s multiple comparison test, *P* ═ 0.0007, Figure 4C). *PU.1* mRNA and *FOXO1* mRNA relative levels were also upregulated in PBMCs with AREG stimulation (one-way ANOVA followed with Tukey tests, *P* < 0.05, Figure 4D and 4E), and anti-HIF-1α treatment suppressed AREG-induced *PU.1* mRNA and *FOXO1* mRNA relative levels (Tukey tests, *P* < 0.05, Figure 4D and 4E). Exogenous AREG enhanced EGFR secretion (median 60.83 [IQR 24.89, 80.98] pg/mL vs median 39.50 [IQR 26.47, 56.09] pg/mL, Kruskal–Wallis test followed with Dunn’s multiple comparison test, *P* ═ 0.0049, Figure 4F) and *EGFR* mRNA relative levels (1.06 ± 0.08 vs 0.97 ± 0.10, one-way ANOVA followed with Tukey test, *P* ═ 0.023, Figure 4G) in PBMCs as compared to cells without exogenous AREG. However, anti-HIF-1α administration did not affect EGFR expression, neither at protein nor at the mRNA level (Dunn’s multiple comparison test or Tukey test, *P* > 0.05, Figure 4F and 4G). AREG also promoted HIF-1α production (median 58.93 [IQR 45.36, 95.54] pg/mL vs median 39.29 [IQR 33.39, 66.79] pg/mL, Kruskal–Wallis test followed with Dunn’s multiple comparison test, *P* ═ 0.013, Figure 4H) and *HIF-1*α mRNA relative levels in PBMCs (1.05 ± 0.10 vs 0.98 ± 0.10, one-way ANOVA followed with Tukey test, *P* ═ 0.029, Figure 4I). Anti-HIF-1α administration strongly suppressed HIF-1α expression (median 47.71 [IQR 38.65, 73.43] pg/mL, Dunn’s multiple comparison test, *P* ═ 0.027, Figure 4H), but did not affect *HIF-1*α mRNA relative levels in PBMCs (1.05 ± 0.06, Tukey test, *P* ═ 0.928, Figure 4I).

## Discussion

To the best of our knowledge, this is the first report regarding the IL-9-secreting T cell regulation in IM patients. We found that IM patients had elevated circulating IL-9 levels as well as increased peripheral Th9 and Tc9 cells. Similarly, transcription factors, PU.1 and FOXO1, were also upregulated in IM patients. Moreover, AREG and HIF-1α were elevated, which correlated with Th9 cells percentages in IM patients. Importantly, AREG induced EGFR expression, which further mediated Th9 and Tc9 cell differentiation in PBMCs from IM patients. AREG/EGFR-induced IL-9 production by CD4^+^ and CD8^+^ T cells was abrogated by HIF-1α inhibition. The current data suggests that the AREG/EGFR/HIF-1α signaling pathway might be essential for IL-9 production by T cells and contributes to the increased Th9 and Tc9 differentiation in IM patients.

IL-9 can be produced by different cells, but Th9 cells Tc9 cells are the major sources of IL-9. Naïve CD4^+^ T cells can differentiate into Th9 cells in the presence of IL-4 and TGF-β1 [28]. Tc9 cells can differentiate from CD8^+^ T cells in a Th9 cell-mediated microenvironment [29], and express very low levels of cytotoxic molecules but secrete a large amount of IL-9 [30]. IL-9 and IL-9-secreting cells serve as double-edged swords in tumor immunity with both pro-tumorigenic and anti-tumorigenic role in cancer development. IL-9 promotes tumor progression in hematological tumors through its lymphocyte growth factor activity [31, 32]. In contrast, IL-9 always plays an anti-tumor function in solid tumors via activating innate and adaptive immune responses [33, 34]. Similarly, controversy remained as to the function of IL-9-secreting T cells during acute and chronic infection. The proportion of Th9 cells was increased in acute phase of visceral leishmaniasis and declined following effective therapies [13]. The frequency of Tc9 cells, but not Th9 cells, was increased in both acute and chronic *Helicobacter pylori*-induced gastritis, but IL-9 was elevated only in chronic active gastritis patients [35]. Experimental *Trypanosoma cruzi* infection induced elevation of Th9 and Tc9 cells in the splenocytes during chronic phase, leading to increased cardiac IL-9 levels compared with uninfected mice [14]. However, in chronic hepatitis B and hepatitis B-related hepatocellular carcinoma, peripheral and liver-infiltrating non-specific and virus-specific Th9 cells, but not Tc9 cells, were reduced [36, 37]. Herein, we found that peripheral Th9 and Tc9 cells were strongly increased in IM patients, along with the elevation of plasma IL-9 and transcription factors for IL-9-secreting T cells. This was consistent with the findings in acute infection [13]. EBV-encoded small RNA was an autocrine growth factor for EBV-infected T cells through induction of IL-9 secretion, indicating that EBV might not only affect the pathogenesis of EBV-associated diseases but also directly contribute to Th9 and Tc9 differentiation [38, 39]. IL-9 played a protective role against *Helicobacter pylori* and helped limit infection in mouse model [40]. Thus, the elevation of Th9 and Tc9 cells might be directly induced by EBV in IM patients.

AREG is one of the ligands for EGFR and is found to be expressed in Th9 cells. However, other common EGFR ligands such as EGF and TGF-α could not be detected in Th9 cells, suggesting a potential regulatory role of AREG/EGFR axis in Th9 differentiation and function [21]. Our present results revealed the elevation of circulating EGFR and AREG in IM patients. EGFR phosphorylation is activated through different signal pathways, resulting in cellular proliferation, differentiation, and survival [41]. Roy et al. [21] showed that abrogation of EGFR signaling repressed only IL-9 expression without affecting the induction of other cytokines in Th9 cells. We found that EGFR inhibition suppressed the proportion of Th9 and Tc9 cells IL-9 production, and transcription factor expression in PBMCs from IM patients, indicating that EGFR signaling is functionally essential for the differentiation of Th9 and Tc9 cells in IM patients. AREG plays a pivotal role in mediating effector and regulatory activities of Th2 and FoxP3^+^ regulatory T cells (Tregs) [42, 43]. Th9 cells share gene program closer to Th2 and Tregs, indicating the potential involvement of AREG in Th9 cells. Previous study identified that AREG augmented Th9 cell differentiation, and EGFR-induced IL-9 secretion by CD4^+^ T cells was notably impaired in AREG knockout mice [21]. Our present data showed that elevated AREG was positively correlated with Th9 and Tc9 cells in IM patients. Importantly, exogenous AREG stimulation of PBMCs from IM patients promoted IL-9-secreting CD4^+^ and CD8^+^ T cell differentiation. This process was accompanied by elevation of EGFR expression, which was consistent with the findings in physiological condition [21]. Taken together, upregulation of AREG/EGFR axis mediated Th9 and Tc9 differentiation in IM patients. The potential mechanisms for AREG/EGFR regulation of IL-9 production still need further elucidation.

The downstream pathways of EGFR signaling are also involved for triggering Th9 cell differentiation [21]. EGFR activation induced HIF-1α, which mediated resistance to anoikis-like cell death under lipid-rafts/caveolae-disrupting stress [44]. EGFR-induced phosphorylation of different pathways contributed to HIF-1α signaling loop, regulated glucose metabolism in pancreatic cancer [45], and promoted hepatocellular carcinoma progression [46, 47]. Roy et al. [21] revealed that EGFR-HIF-1α axis contributed to Th9 cell differentiation. In this study, we found that HIF-1α was upregulated in IM patients and correlated with Th9 cells but not with Tc9 cells. The suppressive function of Th9 and Tc9 cells due to EGFR inhibition was accompanied by downregulation of HIF-1α, suggesting the involvement of HIF-1α in EGFR-mediated IL-9 production by T cells. Furthermore, exogenous AREG stimulation also induced HIF-1α expression. Neutralization of HIF-1α dampened AREG-mediated Th9 and Tc9 differentiation without influencing EGFR expression. This indicated that AREG/EGFR axis-induced IL-9 secretion in IM patients was dependent on HIF-1α production. Thus, AREG/EGFR/HIF-1α signaling pathway contributed to Th9 and Tc9 response and might take part in the pathogenesis of IM.

## Conclusion

In summary, Th9 and Tc9 cells were upregulated in IM patients. The elevation of IL-9 might be essential for controlling acute EBV infection in IM patients. AREG/EGFR/HIF-1α signaling pathway regulated Th9 and Tc9 differentiation, which might contribute to the pathogenesis of IM and serve as one of the therapeutic targets for the treatment of IM.

**Conflicts of interest:** Authors declare no conflicts of interest.

**Funding:** This work was supported by the grant from SPPH Incubator Fund for Development of Science and Technology (2021YJY-19), SPPH Foundation for Development of Science and Technology (2021BJ-26), Xi’an Foundation for Development of Science and Technology [20YXYJ0009(11)], International Science and Technology Cooperation Projects of Shaanxi Province (2022KW-14), and Scientific and Technological Innovation Team of Shaanxi Province (2021TD-40).

## Supplemental Data

**Figure S1.**
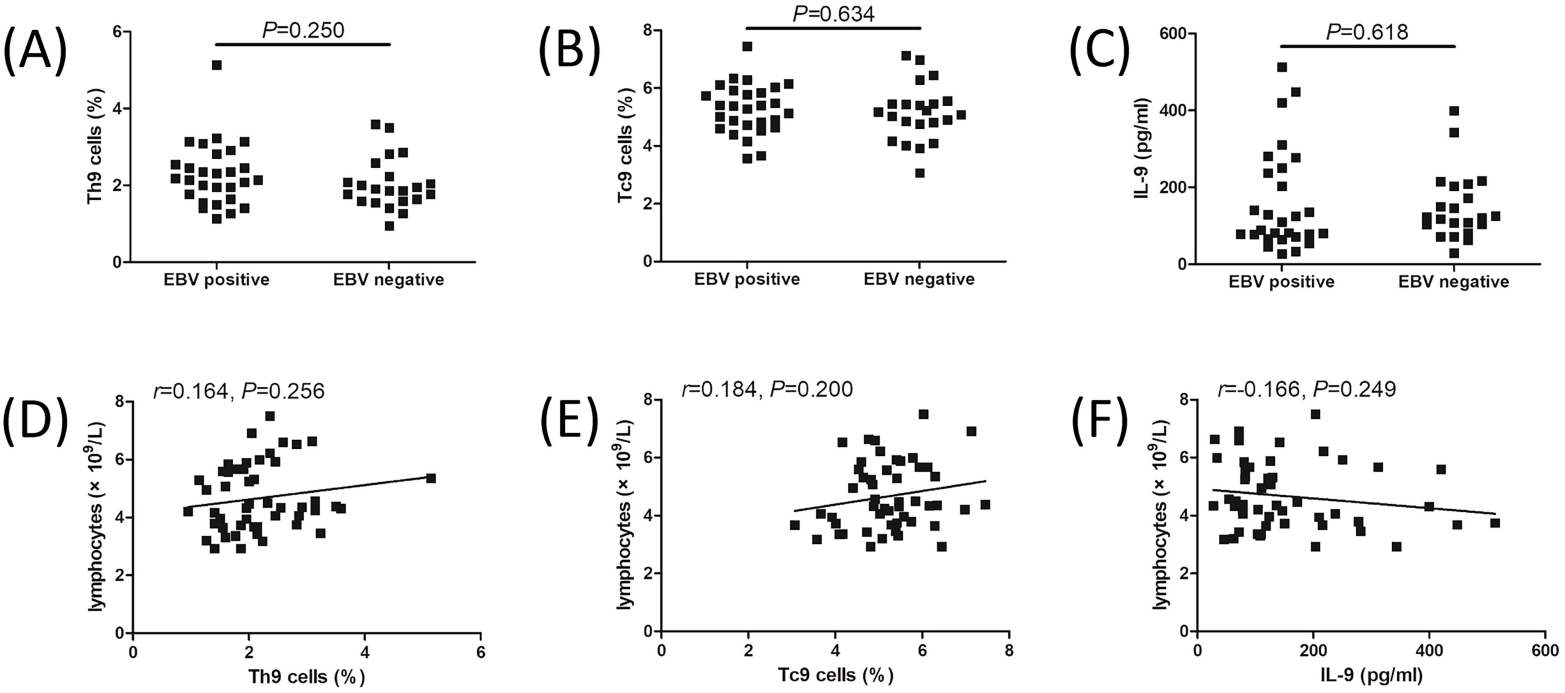
**Th9 and Tc9 cell percentage, and IL-9 levels in IM patients.** (A) Th9 percentage was compared between EBV positive and EBV negative IM patients. (B) Tc9 percentage was compared between EBV positive and EBV negative IM patients. (C) Plasma IL-9 levels were compared between EBV positive and EBV negative IM patients. Student’s *t* test or Mann–Whitney test was used for comparison. (D) Correlation between lymphocyte count and Th9 cell percentage in IM patients. (E) Correlation between lymphocyte count and Tc9 cell percentage in IM patients. (F) Correlation between lymphocyte count and plasma IL-9 levels in IM patients. Pearson or Spearman correlation analysis was performed for correlation analysis. EBV: Epstein–Barr virus; IM: Infectious mononucleosis; IL-9: Interleukin 9; IM: Infectious mononucleosis; Th9: T helper 9 cells; Tc9: T cytotoxic 9 cells.
